# Spontaneous Jejunal perforation in coeliac disease: diagnostic dilemma and navigating treatment beyond gluten-free diet in the absence of refractory disease

**DOI:** 10.1093/omcr/omae210

**Published:** 2025-03-20

**Authors:** Muhammad Hafiz Kamarul Bahrin, Hidayatul Nabila Rosaidi, Ammar Mohd Amin, Mirza Faisal Anwar Baig, Martyn Dibb

**Affiliations:** Gastroenterology, Nottingham University Hospitals, Derby Road, Nottingham NG7 2UH, United Kingdom; Internal Medicine, Royal Derby Hospital, Uttoxeter Road DE22 3NE, United Kingdom; Internal Medicine, Sherwood Forest Hospitals NHS Trust, Mansfield Road, Sutton-In-Ashfield NG17 4JL, United Kingdom; Gastroenterology, Nottingham University Hospitals, Derby Road, Nottingham NG7 2UH, United Kingdom; Gastroenterology, Nottingham University Hospitals, Derby Road, Nottingham NG7 2UH, United Kingdom

**Keywords:** Gastroenterology, General Surgery

## Introduction

Coeliac disease (CD) is relatively common in the West, affecting up to 1% undergoing serological screening and 0.6% histologically confirmed diagnosis of the population [1]. The pathophysiology involves a complex interplay between genetic susceptibility, environmental, and immunologic factors, resulting in chronic inflammation. 95% of people with CD exhibits the HLA-DQ2 and HLA-DQ8 genes which are crucial for presenting gluten-derived peptides to immune cells. The end result is activation of CD4+ T-helper cells in the lamina propria. These cells release inflammatory cytokines such as interferon-gamma that drive chronic inflammation and ultimately villous atrophy [2].

While treatment is mainly conservative and mandatory avoidance of gluten-containing diet, a small proportion fails to response to this measure, leading to progressive disease and refractory malabsorption. This is called the refractory CD (RCD). Diagnosis of this requires absolute proof that Gluten-Free Diet (GFD) has been adopted for at least 12 months, apart from exclusion of other potential causes that may mimic CD [3].

RCD is categorised into type 1 and type 2, based on the presence of aberrant intraepithelial lymphocytes, with the latter carrying a worse prognosis. Most cases of CD associated Ulcerative Jejunitis (UJ), a chronic immune-mediated deep ulceration within the jejunum is found coexisting with type 2 RCD. Very few presents with UJ alone with no RCD association.

## Case description

Our case describes a 34 years old gentleman, admitted en-route into the hospital with acute severe abdominal pain and vomiting upon landing at a local airport. He was mobile, independent and has no known medical or surgical history. He smokes about 10 cigarettes per day and drinks alcohol only occasionally. He works as a sous-chef at a restaurant 50 miles away from the admitting hospital, which is also where he usually resides.

Upon interrogation, he informed us that he was previously investigated by the local GP for 1 year history of weight loss and occasional loose stool. The biochemistry tests were unremarkable, although, his serum IgA anti-TTG was reported positive at 128 CU (4 months prior to travel). He was commenced on GFD whilst awaiting confirmatory test in the form of Oesophagogastroduodenoscopy (OGD) and duodenal histology. Unfortunately, due to multiple factors, he had not yet accessed this test prior to his travel abroad.

On examination, he had a thin and lean body size with a body mass index (BMI) of 16.3 kg/m^2^. His blood tests on arrival to the emergency department were shown in Table 1. HLA DQ-2 and HLA DQ-8 were positive.

**Table 1:** Baseline blood test results on admission. The prominent findings were microcytic anaemia, raised CRP which may have resulted from active inflammation and perforation, and raised lactate due to developing acute peritonitis.

**Blood test**	**Value**	**Normal Range**
**Hb (g/l)**	93.0	120–155
**White Cell Count (**× **10^9/l)**	13.3	4.0–11.0
**Platelet (**× **0^9/l)**	450	150–400
**Mean Corpuscular Volume (fl)**	76.7	81.0–99.0
**Ferritin (ng/ml)**	1283	<15
**C-Reactive Protein (mg/l)**	220	<1
**Serum Lactate (mmol/l)**	4.6	<1.0
**Endomysial Antibody**	positive	

**Figure 1:**
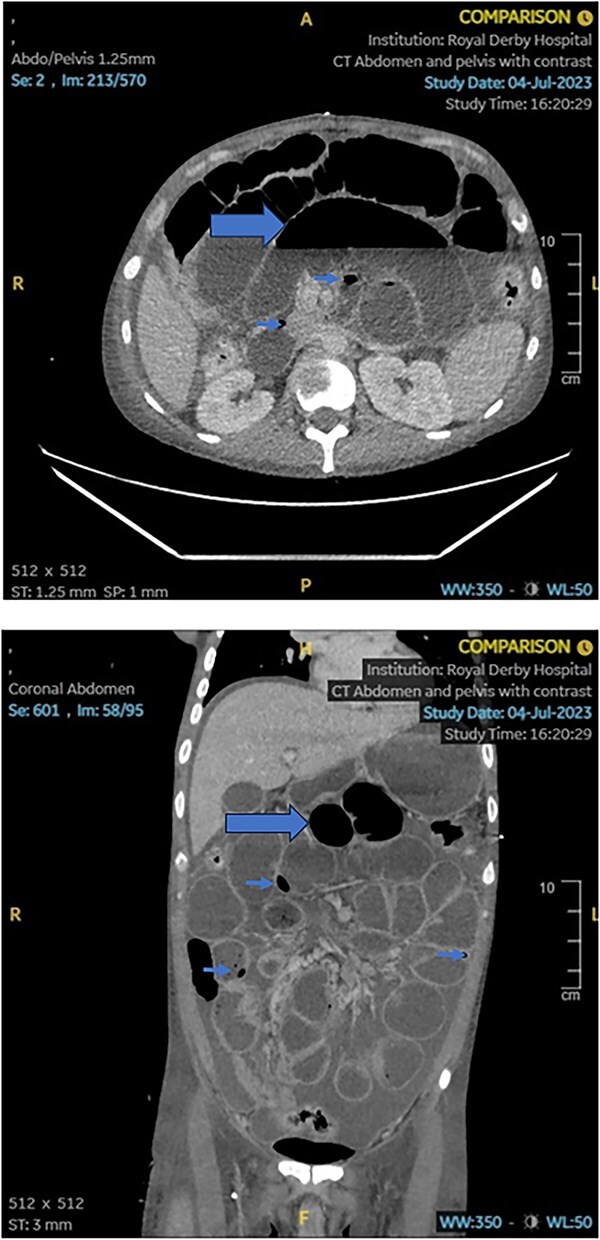
Axial and sagittal views of the patient’s initial CT scan demonstrating free intraperitoneal and intramural air (arrowheads), as well as small bowel dilatation (arrow) in keeping with an acute small bowel perforation. Free intraperitoneal fluid can also be seen.

A contrast-enhanced Computed Tomography of abdomen and pelvis (CT AP) was promptly performed. This demonstrated ‘*free intraperitoneal fluid and air, intramural air and bowel dilatation’ raising suspicion of a small bowel obstruction, perforation and peritonitis*. The changes are demonstrated on the images below (Fig. 1):

An emergency laparotomy was performed, and the perforated site of the jejunum was located. A limited jejunal resection was carried out, leaving behind 150 cm distal to the DJ flexure (afferent) and 4 m of small bowel distal to the resection site (efferent). Subsequently, both jejunal ends were exteriorised to form a double-barrelled ileostomy, with an intention to anastomose in the future. Parenteral nutrition was commenced.

**Figure 2:**
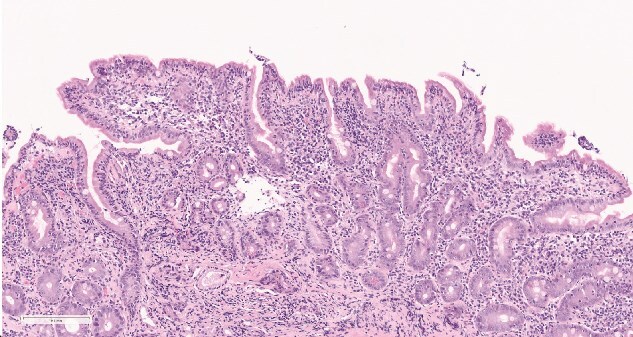
Marsh 3b coeliac disease characterised by intraepithelial lymphocytosis, crypt hyperplasia and subtotal villous atrophy. Active disease is indicated by histological features in keeping with marsh 3a classification and above (any degree of villous atrophy).

His recovery however, was hampered by intermittent episodes of frank blood passage via the ileostomy, associated with significant drop in haemoglobin and fluid-responsive hypotension. An urgent post operative OGD was undertaken which demonstrated no stigmata of upper GI bleed to D3. Duodenal biopsies were obtained and was reported as ‘*Intraepithelial lymphocytosis (IELs), partial villous atrophy and crypt hyperplasia in keeping with coeliac enteropathy, Marsh 3b classification* (Fig. 2)’.

**Figure 3:**
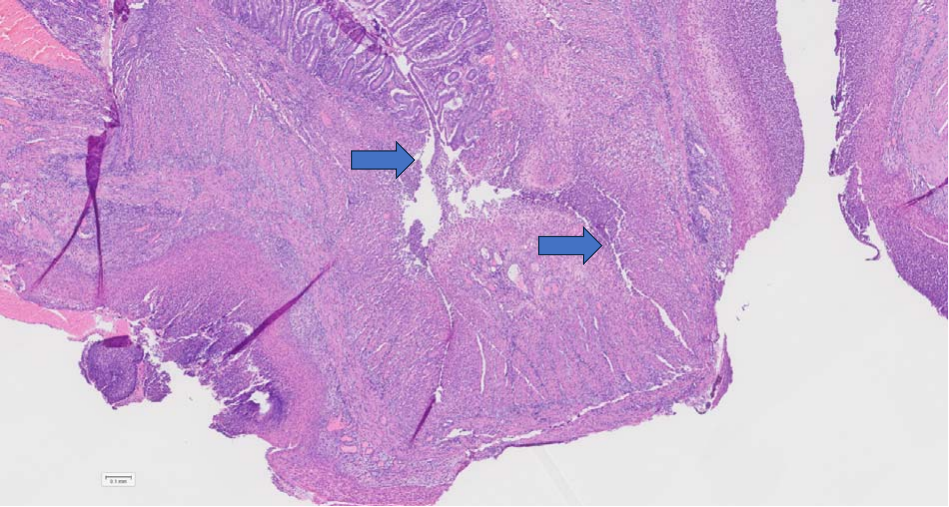
Resected bowel specimen histology demonstrating deep fissuring ulceration and perforation (arrow), in the background of typical coeliac enteropathy changes.

His resected bowel specimens were also reviewed by 4 histopathologists, 2 of which, were specialised Gastro-Intestinal histopathologists from the tertiary centre of the region. This was reported as:

‘*The small bowel sections show extensive deep fissuring ulceration with peritonitis in keeping with perforation. There is however no evidence of chronic transmural inflammation to suggest Crohn’s disease, and the surrounding bowel does not look particularly ischaemic, nor is there evidence of a primary vasculitis. The surrounding bowel does show varying degrees of villous atrophy and there is also increased intraepithelial lymphocytes, although not as many as in the duodenum, but this is in keeping with CD’* (Fig. 3).

T-Cell receptor gene re-arrangement did not demonstrate aberrant intraepithelial lymphocytes. Immunohistochemistry and flow cytometry showed normal surface expression of CD3+/CD8+ IELs. Staining for Cytomegalovirus (CMV) and Whipple’s disease were also negative.

A presumed diagnosis of UJ associated with aggressive CD was reached by the multidisciplinary team (MDT). Due to lack of evidence to support type 2 RCD, this was thought to be unlikely.

## Further issue

In view of ongoing malnutrition and GI bleed, a trial of glucocorticoid therapy was experimented following an in-depth risk and benefit discussion with the patient and his next of kins, explicitly highlighting the risk of septicaemia and failure of surgical wound healing, should this attempt fail and a salvage surgical intervention is required.

To our relief, our patient made a remarkable improvement following the glucocorticoid therapy trial, evidenced by increasing body weight by 20% within 1 week and reduced dependency on blood transfusion to once a week.

## Outcome

We switched his glucocorticoid treatment to oral form after 2 weeks of sustainable weight gain and a stable level of haemoglobin. He was also commenced on Azathioprine (AZA) treatment at 100 mg once a day to be taken long term, alongside a tapering course of glucocorticoid and strict advice on GFD on discharge.

Following up his case 12 months down the line, he had undertaken duodenal biopsies at his local hospital at 6 months post discharge. This demonstrated persistent IELs, crypt hyperplasia and raised anti-TTG to 100 CU (was expected to reduce by half if treatment-responsive) despite ongoing course of AZA. He was commenced on 8 weekly dosing of infliximab infusion to downstage the disease prior to reversal surgery. Tofacitinib was also suggested by the local MDT, although the former was selected due to patient’s choice. Having said so, his BMI is relatively stable.

## Discussion

CD, whilst not particularly rare among the Western population, remains an uncharted territory among general clinicians. Strict adherence to GFD is mandatory at the earliest opportunity and involvement of the dietitian service is paramount. Symptomatic remission is expected in just a few weeks after a GFD, while histological remission may take 6 to 18 months to achieve [4]. When there are persistent symptoms and failure of weight gain despite 12 months trial of GFD, one is said to have developed refractory CD [3, 5].

To date, RCD remains a diagnosis of exclusion, after potential mimics such as IBD or colorectal cancer have been ruled out via invasive tests such as ileocolonoscopy or enteroscopy.

### Diagnostic modalities for RCD

The main diagnostic workup considers the use of endoscopy and histopathology. It is of interest to note that while the European Society of Gastrointestinal Endoscopy (ESGE) does not support the use of small bowel capsule endoscopy (CE) to diagnose CD due to its relatively low sensitivity and specificity, there is a role for its use in the initial assessment of RCD. The main limitations being its low diagnostic yield for complications such as EATL and UJ; and the risk of capsule retention from small bowel strictures [6].

That being said, there are multiple case reports of UJ diagnosed via CE such as the ones documented by Jasleen *et al.* and Le mouel *et al.* [7, 8]. In both cases, characteristic villous atrophy, oedema and diffuse ulceration were revealed on the CE. The former also describes an inflammatory stricture of the jejunum. Both were followed up by a subsequent biopsy via enteroscopy.

The histopathological features found in CD commonly include villous atrophy, crypt hyperplasia and an IEL count of more than 25/100. In suspected RCD, further histological testing with immunostains is required to differentiate between type 1 and 2 RCD. The presence of aberrant IEL immunophenotype especially with a reduced CD3, CD4 and CD8 expression favours a diagnosis of RCD2. Recently the use of a diagnostic biomarker, NKp46, has been proposed as it is found to be significantly more expressed in type 2 RCD [9].

UJ rarely presents without type 2 RCD but is not impossible. This happens due to chronic idiopathic ulceration affecting the small intestine and localisation is common in ileum or jejunum, rarely in colon. Histological features typically include full thickness mucosal ulceration, on a background of CD changes. There may also be coexistent chronic inflammation, fibrosis, and muscular hypertrophy; responsible for stricture formation [10].

### Medical treatment of ulcerative Jejunitis in RCD

RCD lacks a standardized treatment regimen due to its rarity. Management of RCD type 1 typically improves with strict nutritional and pharmacological interventions, while RCD type 2 is more challenging, with limited trial data guiding treatment. Steroids and steroid-sparing agents, such as azathioprine, are commonly used. Nutritional support is also crucial, particularly for patients with severe malnutrition or weight loss, where parenteral nutrition is recommended to support intestinal recovery [11]. Hospitalisation may be necessary for severe cases to monitor GFD adherence.

A retrospective study by Nasr et al. (2015) showed that a combination of prednisolone and azathioprine improved prognosis in RCD type 2, with 53% of patients achieving histological recovery [12]. For those intolerant to these drugs, alternatives like thioguanine, methotrexate, or mycophenolate mofetil were used. Other therapies include immunosuppressants like cyclosporin and biologics such as infliximab. Cladribine has shown promise but poses lymphoma risks [13]. Additionally, autologous hematopoietic stem cell transplantation has been explored, with significant symptom improvement in most patients, though long-term monitoring remains essential [14].

### Janus-kinase (JAK) inhibitor use in RCD

A recent multi-national open-label prospective pilot study by Dieckman et al. (2024) (Netherlands and Germany) proposed JAK inhibitor as a potential targeted therapy for type 2 RCD with no additional adverse effect profile as outlined in the current literature [15]. 6 patients were followed up over a 12-weeks period following commencement of Tofacitinib course and major improvement in villous atrophy was noted in 4 patients. Additionally, 2 patients with UJ showed complete ulceration healing in follow-up CEs at the end of the study period. Cautious interpretation was advised by the authors due to small study sample.

## Conclusion

This case highlights the complexities of managing CD related UJ without clear evidence of RCD. After emergency surgery and nutritional support, the patient responded well to glucocorticoid therapy, showing significant improvement. Given the rarity of this condition, treatment was guided by limited literature and multidisciplinary input. The case emphasizes the need for personalized treatment, early immunosuppressive therapy, and ongoing monitoring to prevent complications. It also adds valuable insights into the management of this rare condition, underscoring the importance of long-term follow-up.
